# 

**Published:** 1831-05

**Authors:** 


### MONTHLY LIST OF MEDICAL BOOKS.

[*Medical Works cannot be entered on this List unless a copy be sent for the purpose, the titles of Books having frequently been sent to us as published which have not been published for weeks, or even months, after.*]

A Critical Exposition of Phrenology; being a Lecture delivered at the Philosophical Institution, Birmingham, by Charles Covey, Member of the Royal College of Surgeons; Surgeon to the Birmingham Dispensary, &c.—Birmingham. 1831.

Lectiones Celsianæ et Gregorianæ; or, Lessons in Celsus and Gregory: consisting of Passages from those Authors, syntactically arranged, with copious Observations explaining the Difficulties of Construction, and a Lexicon of the Words. To which is added, a succinct and comprehensive Grammar, written and adapted for the Work. For the Use of Medical Students. By William Cross, Teacher of the Classics and Medical Latin.—12mo. pp. 169. London: Wilson, 1831.

The Surgical Anatomy of some of the principal Vessels of the Head.

For this elegant and useful Plate the profession is indebted to Mr. Cocks. A clear description of the parts illustrated is given in the margin. The operations of opening the jugular vein, of exposing and tying tbe Carotid Arteries, and of bleeding in the Temporal Artery, are particularly pointed out. This Plate, together with the accurate verbal description that accompanies it, will be studied with much advantage by young surgeons and students.

The Effects of the principal Arts, Trades, and Professions, and of Civic States and Habits of Living, on Health and Longevity: with a particular Reference to the Trades and Manufactures of Leeds; and Suggestions for the Removal of many of the Agents whieh produce Disease, and shorten the Duration of Life. By C. Turner Thackrah.—8vo. pp. 126. London: Longman, 1831.

Letters to a Mother on the Watchful Care of her Infant.—12mo. pp. 144. London: Seeley and Burnside, 1831.

A useful book for young mothers to peruse: the author does not address professional readers.

An Essay on the Influence of Temperament in Modifying Dyspepsia or Indigestion. By Thomas Mayo, m.d., Physician in Ordinary to His Royal Highness the Duke of Sussex, &c.—8vo. pp. 144. London: Fellowes, 1831.

Treatise on the Excision of Diseased Joints. By James Syme, f.r.s.e., Surgeon of the Edinburgh Surgical Hospital, Lecturer on Surgery, &c. &c.—8vo. pp. 163; plates. Longman, 1831.

**Illustrations of Mr. S. Cooper's Surgical Dictionary: published Monthly. Parts VII. to XII. (inclusive.)**

We have before recommended these Illustrations to the attention of surgical students. There are in surgery many practical facts, of which verbal descriptions can hardly convey a clear comprehension to the mind of the student; and a reference to this work will remove such difficulties, and render his task more agreeable, because it will more certainly be profitable.

